# Synchronized in-gap edge states and robust copropagation in topological insulators without magnetic flux

**DOI:** 10.1016/j.fmre.2025.01.005

**Published:** 2025-01-21

**Authors:** Liangcai Xie, Tianyi He, Liang Jin

**Affiliations:** School of Physics, Nankai University, Tianjin 300071, China

**Keywords:** In-gap edge state, Copropagation, Time-reversal symmetry, Topological insulator, Metal-insulator phase transition

## Abstract

Copropagation of antichiral edge states in the metallic phase requires the bulk states as counterpropagating modes. Without the band gap protection, the copropagation along the boundaries is easily scattered into the bulk and the counterpropagation in the bulk is not robust against disorder and defects. Here, we propose a novel time-reversal symmetric topological insulator holding the synchronized in-gap edge states. To prevent the participation of bulk states, we introduce the time-reversal symmetry to detach the edge states from the bulk band, then the robust copropagation is realized through widening the band gap across a metal-insulator transition with anisotropic next-nearest-neighbor couplings. The time-reversal symmetry ensures the in-gap edge states with opposite momenta as the counterpropagating modes. The inversion symmetry protects the quantized polarization as a topological invariant. The synchronized in-gap edge states not only enrich the family of topological edge states, but also provide additional flexibility in the design of reconfigurable topological optical devices. Our findings open a new avenue for the tailored robust wave transport using the in-gap edge states for future acousto-optic topological metamaterials.

## Graphical abstract

**Figure:**
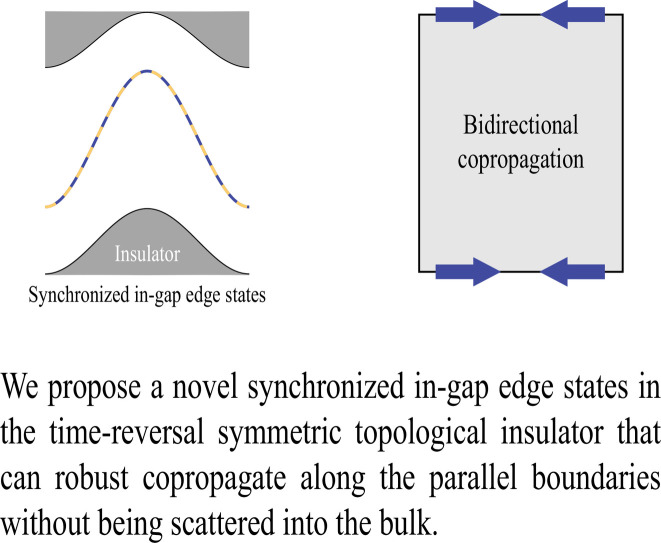

## 1 Introduction

Topological states in topological phases are robust against disorder and defects [1]. In topological insulators, the chiral edge states unidirectionally propagate in the opposite directions along the parallel boundaries. The band gap protects the robustness of chiral edge states. The helical edge states are constituted by a pair of time-reversal symmetric chiral edge states associated with the pseudospin-up and pseudospin-down [2], [3], [4], [5]. The valley is an independent degree of freedom similar to the pseudospin [6], [7], [8]. The time-reversal symmetry of each individual pseudospin is broken in the spin-Hall phase and the inversion symmetry is broken in the valley-Hall phase [9]. The propagations of helical edge states in spin-Hall phase or kink edge states in valley-Hall phase are pinned to the pseudospins or valleys. The helical/kink edge states associated with the same pseudospin/valley along the parallel boundaries are counterpropagating modes for each other.

In topological metals, the antichiral edge states unidirectionally copropagate in the same direction along the parallel boundaries [10]. The band gap is absent and the on-resonant bulk states act as counterpropagating channels for the antichiral edge states [11], [12]. Through reversing the magnetic flux associated with one of the two sublattices, the topological phase changes from insulator to metal and the chiral edge states become antichiral edge states [13], [14], [15]. Without the band gap protection, once the antichiral excitation copropagating along the boundaries is scattered into the bulk by disorder or at the corners, the recovery of copropagation is impossible. An intriguing and important question is whether it is possible to find copropagating edge states that can avoid the participation of bulk states in the realization of copropagation?

**Fig. 1:**
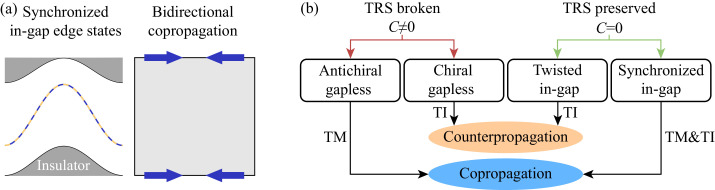
(a) Schematics of the energy band structure for the synchronized in-gap edge states and the associated bidirectional copropagation. (b) Schematic of distinct types of two-dimensional edge states and their propagations. TM stands for topological metal and TI stands for topological insulator.

Here, we find a novel time-reversal symmetric topological insulator that hosts a pair of degenerate in-gap edge states, which synchronously copropagate along the parallel boundaries back and forth against disorder without being scattered into the bulk [Fig. 1(a)]. This is realized by widening the band gap until a metal-insulator phase transition. In the topological insulating phase, the wide band gap provides a strong topological protection, prohibits the counterpropagation in the bulk, and protects the robustness in the copropagation. The time-reversal symmetry ensures the bidirectionality. The inversion symmetry ensures the synchronization. Furthermore, we demonstrate the disorder-immune robust copropagation along the parallel boundaries for the edge state excitation. The novel topological phase, supporting intriguing topological wave transports mediated by the anisotropic long-range couplings, fundamentally differs from the spin-Hall phase, valley-Hall phase, and high-order topological phase. The flux-free setup without breaking the time-reversal symmetry facilitates the experimental realization in topological metamaterials.

The elaboration of our findings is organized as follows. We start by introducing the definitions of gapless and in-gap edge states. Then, we elucidate different types of two-dimensional edge states from the viewpoints of time-reversal symmetry, band structure, and robust propagation dynamics along the boundaries. The features of synchronized in-gap edge states are revealed and highlighted from the demonstrations of time-reversal symmetry breaking/preserving robust counterpropagations/copropagations exemplified in the extended Qi-Wu-Zhang model, which is a square lattice having nontrivial topology only in one direction. Furthermore, we propose a time-reversal symmetric square lattice without magnetic flux holding the topological insulating phase with synchronized in-gap edge states in both directions by using the unbalanced reciprocal next-nearest-neighbor couplings. Finally, we perform numerical simulations to show the robust copropagation of synchronized in-gap edge states along the parallel boundaries in the presence of random disorder.

## 2 Results and discussion

Topological edge states are defined as gapless and in-gap from relative position between the edge states E(k) and bulk bands $\varepsilon_{\pm }\left(k\right)$. The edge states are called gapless if $E\left(k\right)=\varepsilon_{\pm }\left(k\right)$ for certain k in the Brillouin zone (BZ); i.e., the edge states are contacted with two adjacent bulk bands. The edge states are called in-gap if $E\left(k\right)\neq \varepsilon_{\pm }\left(k\right)$ for all k∈BZ; i.e., the edge states are detached from the bulk band. The in-gap edge states spread in the entire BZ. Consequently, the in-gap edge states support *bidirectional* propagation unless they are fully flat. In particular, the edge states are called fully in-gap if $E\left(k\right)\neq \varepsilon_{\pm }\left(k^{\prime }\right)$ for all k, $k^{\prime }\in \mathrm{BZ}$; i.e., the edge states fully reside within the band gap. The gapless and in-gap edge states may exist in the insulators and metals, whereas the fully in-gap edge states only exist in the insulators.

**Fig. 2:**
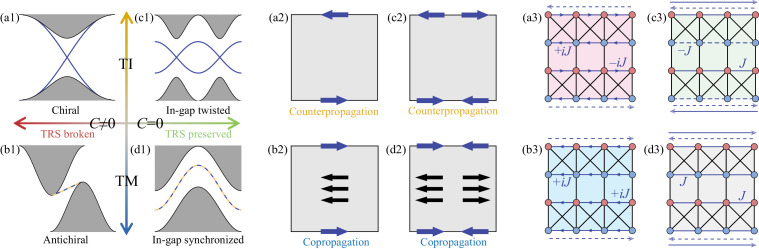
**Schematics of robust propagations of edge states along the parallel boundaries, the associated band structures, and their realizations in the square lattices**. (a) Unidirectional counterpropagation of chiral edge states. (b) Unidirectional copropagation of antichiral edge states. (c) Bidirectional counterpropagation of twisted in-gap edge states. (d) Bidirectional copropagation of synchronized in-gap edge states. In (a) and (b), the time-reversal symmetry breaks and the edge states are gapless. In (c) and (d), the time-reversal symmetry preserves and the edge states are in-gap. The inversion symmetry results in the degeneracy of the in-gap edge states in (d). Copropagation in the insulators is realized after the metal-insulator transition by widening the band gap in (d) as illustrated in Fig. 1(a).

Figure 1(b) concisely summarizes the two-dimensional edge states in the first-order topological phases from the viewpoints of the time-reversal symmetry breaking or preserving, the gapless or in-gap, and the metallic phase or insulating phase. Figure 2 provides the schematics of the band structures, the ways of propagations, and the concrete lattice realizations of different types of edge states. More specifically, the chiral (antichiral) edge states are the gapless edge states associated with the topological insulators (metals) as elucidated in Fig. 2(a1) [Fig. 2(b1)], and the unidirectional counterpropagation (copropagation) along the parallel boundaries is schematically illustrated in Fig. 2(a2) [Fig. 2(b2)] as a consequence of time-reversal symmetry breaking. For the gapless edge states, the metal-insulator phase transition alters the energy band from a topological insulator to a topological metal and the topological edge states alter from chiral to antichiral. This fundamentally stems from the fact that the edge states are gapless, i.e., the bulk bands and the edge states are touched. This causes the bulk states to participate as the counterpropagating modes of antichiral edge states in a closed loop of propagation dynamics.

To prevent the participation of bulk states, we introduce the time-reversal symmetry to add a backward going channel at the edges. The antichiral edge states are altered into the in-gap edge states, i.e., the bulk band and the edge states are detached from each other. We emphasize that although the time-reversal symmetry ensures a zero Chern number, the topological phase might still be nontrivial. Time-reversal symmetric insulators with nontrivial topology may support the twisted in-gap edge states [Fig. 2(c1)]. Interestingly, we discover novel time-reversal symmetric topological insulators supporting the synchronized in-gap edge states [Fig. 2(d1)]. The time-reversal symmetry guarantees the spectrum at the opposite momenta having the same energy and opposite velocities. Thus, the in-gap edge states include both the forward-going and backward-going edge modes, and the boundaries support bidirectional light flow. In Fig. 2(c2) [Fig. 2(d2)], the edge state excitations with the same momentum in the time-reversal symmetric insulators (metals) counterpropagate (copropagate) along the parallel boundaries in the opposite (same) directions with the same velocity.

In time-reversal symmetric topological phases, we emphasize that the copropagation may appear in both the metallic [Fig. 2(d1)] and insulating phases [Fig. 1(a)]. For the copropagation of in-gap edge states in the metallic phase, the edge and bulk states both participate as counterpropagating modes [Fig. 2(d1)]. Widening the energy bands illustrated in Fig. 2(d1) alters the metallic phase into the insulating phase, meanwhile the in-gap edge states are altered into the fully in-gap edge states illustrated in Fig. 1(a). For the copropagation of fully in-gap edge states in the insulating phase, only the edge states participate as the counterpropagating modes. The band gap protects that the edge and bulk states are off-resonant, and the copropagation of fully in-gap edge states remains robust without being scattered into the bulk. The excitation of in-gap edge states on the parallel boundaries with the same momenta realizes the robust copropagation in the same directions as protected by the inversion symmetry.

Now, we consider the concrete lattices holding the topological phases and edge states discussed previously in this section. The Haldane model is a honeycomb lattice with nonreciprocal next-nearest-neighbor couplings, which breaks the time-reversal symmetry. The standard Haldane model with identical magnetic flux associated with its two sublattices holds the topological insulating phase [1]. The modified Haldane model with opposite magnetic fluxes associated with its two sublattices holds the topological metallic phase [10]. The nonzero Chern numbers predict the chiral and antichiral edge states in both cases. To generate a time-reversal symmetric topological phase, a simple consideration is the removal of magnetic fluxes in the Haldane model. The standard Haldane model without magnetic flux is a hexagonal lattice with reciprocal next-nearest-neighbor couplings. Unfortunately, such a time-reversal symmetric hexagonal lattice *cannot* be tuned into an insulator due to the inseparable energy bands. To propose the time-reversal symmetric topological phases with separable energy bands with a band gap and the in-gap edge states, we alternatively consider the extended Qi-Wu-Zhang model, which is a square lattice with the reciprocal next-nearest-neighbor couplings uniformly presented in one direction and alternatively presented in the other direction [16].

The time-reversal symmetry breaks in both the square lattices shown in Fig. 2(a3) and (b3). Figure 2(a3) is the square lattice having the insulating phase with the band structure shown in Fig. 2(a1) and supporting the chiral edge states. In this configuration, the nonreciprocal couplings along the top and bottom boundaries are opposite and the magnetic fluxes associated with the upper and lower triangles are identical. Figure 2(b3) is the square lattice having the metallic phase with the energy band structure shown in Fig. 2(b1) and supporting the antichiral edge states. In this configuration, the nonreciprocal couplings along the top and bottom boundaries are identical and the magnetic fluxes associated with the upper and lower triangles are opposite. The time-reversal symmetry is preserved in both the square lattices shown in Fig. 2(c3) and (d3). Figure 2(c3) is the square lattice having the insulating phase with the band structure shown in Fig. 2(c1) and supporting the twisted in-gap edge states. In this configuration, the reciprocal couplings along the top and bottom boundaries have opposite signs. Figure 2(d3) is the square lattice having the metallic phase with the band structure shown in Fig. 2(d1) and supporting the synchronized in-gap edge states. In this configuration, the reciprocal couplings along the top and bottom boundaries have identical sign. The synchronized in-gap edge states in Fig. 2(d1) are protected by the inversion symmetry. The metallic phase alters to the insulating phase via widening the band gap; then, the topological insulators holding robust copropagation of in-gap edge states without being scattered into the bulk are realized.

The time-reversal symmetric topological phases have a zero Chern number, but there exists another topological invariant to characterize the band topology [17]. Notably, the time-reversal symmetry and inversion symmetry jointly protect the vanishing of Berry curvature in the entire BZ, and the Berry connection does not have any singularity. In this situation, the two-dimensional Zak phase, known as the polarization, is valid for topological characterization

(1) $$P={\left(2\pi \right)}^{-2}\int_{\mathrm{BZ}}A\left(k_{x},k_{y}\right)dk_{x}dk_{y},$$

where $A\left(k_{x},k_{y}\right)=-\langle \psi \left(k\right)|i\nabla_{k}|\psi \left(k\right)\rangle$ is the Berry connection and |ψ(k)〉 is the Bloch wave function. The two components of polarization $P=\left(P_{x},P_{y}\right)$ describe the topologies of the one-dimensional projection lattices. $P_{x}\neq 0$ and $P_{y}\neq 0$ indicate the existence of in-gap edge states under the open boundary condition (OBC) in the x- and y-direction, respectively.

Although the extended Qi-Wu-Zhang model in the configuration Fig. 2(d3) realizes a topological insulating phase holding the synchronized in-gap edge states on the top and bottom boundaries, the band topology on the other direction is trivial, and the edge states are absent on the left and right boundaries. A topological insulating phase supporting the robust copropagation in both directions greatly benefits the robust light steering in topological metamaterials. This motivates us to further search for a configuration with more flexible band topology in both directions.

**Fig. 3:**
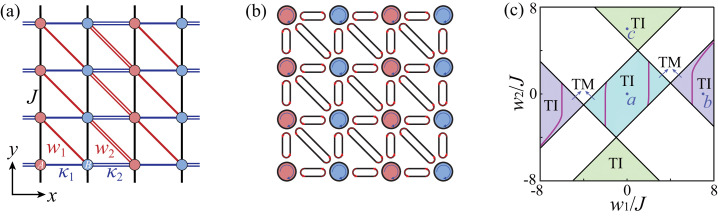
(a) Schematic of the two-dimensional time-reversal symmetric square lattice. (b) Schematic of the coupled-resonator realization. The ring resonators in red (blue) are the sites A (B), and the other stadium resonators are the linking resonators. The blue (red) arrows in the resonators indicate the clockwise (counterclockwise) mode. (c) Phase diagram for $\kappa_{1}=J,\kappa_{2}=4J$.

Based on the configuration in Fig. 2(d3) by destroying the balanced next-nearest-neighbor couplings and maintaining the staggered feature in one direction, we propose a time-reversal symmetric square lattice without magnetic flux as shown in Fig. 3(a). Notably, the configuration in Fig. 3(a) also has the $C_{2}$ symmetry and is easy to realize in optical waveguides [4], resonators [18], [19], and photonic crystals [20], [21]. The flourishing development of topological photonics has spurred experimental simulations of topological systems, providing researchers with platforms to explore novel photonic topological phenomena. Resonators are widely used to simulate topological systems due to their mode stability, high quality factor and integrability. The nonreciprocal couplings are absent in the time-reversal symmetric two-dimensional square lattice. Then, the coupled waveguide arrays, acoustic, electric, and mechanical systems are suitable platforms for the investigation of time-reversal symmetric topological phases without any magnetic flux. In these physical systems, the couplings among the lattice sites are created via directly linking the sites. Thus, the next-nearest-neighbor couplings are unrelated to the nearest-neighbor couplings in the square lattice, and all the couplings are independently tunable.

Figure 3 (b) is the schematic of the coupled resonator realization of the time-reversal symmetric two-dimensional square lattice. The ring resonators are the primary resonators for the sites of the square lattice. The primary resonators in red are the sites A and primary resonators in blue are the sites B. Each resonator supports both the clockwise and counterclockwise modes and the intracavity modes are decoupled. The linking resonators mediate the photons tunneling in the primary resonators and induce the effective couplings between the nearest-neighbor primary resonators and the next-nearest-neighbor primary resonators. The linking resonators and the primary resonators are coupled through their evanescent fields and the coupling strengths depend on the distance between the linking resonators and the primary resonators [2]. The time-reversal symmetric square lattice only has reciprocal couplings. The coupling strength κ is approximately characterized by $\kappa =\kappa_{l}^{2}/\Delta_{l}$, where $\kappa_{l}$ and $\Delta_{l}=\omega_{c}-\omega_{link}$ are the hopping and detuning between the frequency of primary resonators $\omega_{c}$ and the frequency of linking resonators $\omega_{link}$.

Applying the Fourier transformation to the Hamiltonian of the square lattice in the real space, we obtain the Bloch Hamiltonian in the momentum space in the form of

(2) $$h\left(k\right)=d\left(k\right)\cdot \sigma +d_{0}\left(k\right)\sigma_{0},$$

where $\sigma =\left(\sigma_{x},\sigma_{y},\sigma_{z}\right)$ is the Pauli matrix and $\sigma_{0}$ is the identity matrix. The effective magnetic field is $d\left(k\right)=\left[d_{x}\left(k\right),d_{y}\left(k\right),d_{z}\left(k\right)\right]$ with

(3) $$\begin{array}{ccc} d_{x}\left(k\right) & = & \kappa_{1}+\kappa_{2}\cos k_{x}+w_{1}\cos k_{y}+w_{2}\cos\left(k_{x}-k_{y}\right), \end{array}$$

(4) $$\begin{array}{ccc} d_{y}\left(k\right) & = & \kappa_{2}\sin k_{x}+w_{1}\sin k_{y}+w_{2}\sin\left(k_{x}-k_{y}\right), \end{array}$$

(5) $$\begin{array}{ccc} d_{z}\left(k\right) & = & 0. \end{array}$$

The term $d_{0}\left(k\right)=2J\cos k_{y}$ varies the energies of the Bloch bands

(6) $$\varepsilon_{\pm }\left(k\right)=d_{0}\left(k\right)\pm [d_{x}^{2}\left(k\right)+d_{y}^{2}{\left(k\right)]}^{1/2},$$

however, the eigenstates of the Bloch bands are unaffected.

The polarization $P=\left(P_{x},P_{y}\right)$ is associated with the vector field $\left[d_{x}\left(k\right),d_{y}\left(k\right)\right]$ (see Supplementary A). The nontrivial topology P≠(0,0) predicts the edge states in the separable region of the projection spectra (see Supplementary B). The uniform nearest-neighbor coupling J in the vertical direction can open a band gap without destroying the topology. The staggered nearest-neighbor couplings $\kappa_{1}$, $\kappa_{2}$ in the horizontal direction and the anisotropic next-nearest-neighbor couplings $w_{1}$, $w_{2}$ create the nontrivial topology, adjust the edge states, and alter the robust dynamics. We highlight the flexibility of nontrivial topology induced by the anisotropic next-nearest-neighbor couplings. The copropagations in the horizontal and vertical directions can be independently tunable. Topologically nontrivial region in the x-direction characterized by $P_{x}=1/2$ satisfies $|\kappa_{1}+w_{1}e^{ik_{y}}\left|<\right|\kappa_{2}+w_{2}e^{ik_{y}}|$, which predicts the existence of edge states under the OBC in the x-direction. Topologically nontrivial region in the y-direction characterized by $P_{y}=\pm 1/2$ satisfies $|\kappa_{1}+\kappa_{2}e^{ik_{x}}\left|<\right|w_{1}+w_{2}e^{ik_{x}}|$, which predicts the existence of edge states under the OBC in the y -direction. The strong coupling $\kappa_{1}$ ($w_{2}$) destroys (constructs) the topologies in both directions. The strong coupling $\kappa_{2}$ ($w_{1}$) constructs (destroys) the topology in the x-direction and destroys (constructs) the topology in the y-direction. The topological phase transition occurs when the band gap closes at $w_{1}-w_{2}=\pm \left(\kappa_{1}-\kappa_{2}\right)$ and $w_{1}+w_{2}=\pm \left(\kappa_{1}+\kappa_{2}\right)$.

The profile of phase diagram in the parameter space $w_{1}\;-\;w_{2}$ [Fig. 3(c)] depends on the ratio of $\kappa_{1}$ and $\kappa_{2}$. The colored regions are gapped phases. The band gap ensures the separation of the bulk and edge states. The magenta lines indicate the metal-insulator phase transition. In the transition from a topological metal to a topological insulator, the band gap widens without a topological phase transition; and the edge states change from in-gap to fully in-gap, allowing the copropagation without the participation of bulk states as the counterpropagating modes. $w_{1}$, $w_{2}$, and $\kappa_{2}$ increase the band gap to enter topological insulating phase. The white regions are gapless phase with band touching.

**Fig. 4:**
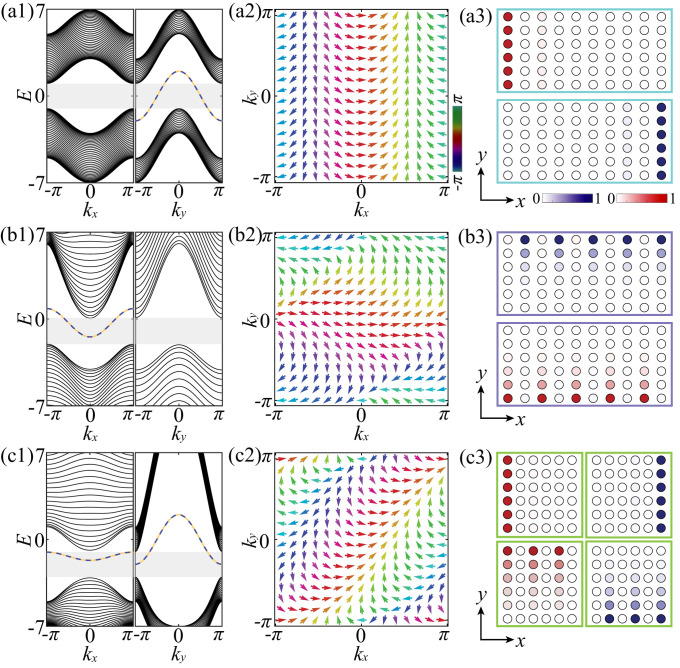
**The projection energy band structure (left panel), the visualized polarization (middle panel), and the edge state distribution (right panel) for** (a) $w_{1}=0,w_{2}=0$, (b) $w_{1}=7J,w_{2}=0$, and (c) $w_{1}=0,w_{2}=6J$ as marked in Fig. 3(C). The angles of the arrows represent $\arctan[d_{y}\left(k\right)/d_{x}\left(k\right)]$ at the momentum $k=(k_{x},k_{y})$. The values of polarization are (a2) $P_{x}=1/2$, $P_{y}=0$, (b2) $P_{x}=0$, $P_{y}=1/2$, and (c2) $P_{x}=1/2$, $P_{y}=-1/2$, respectively.

The polarization in the cyan region is $\left(P_{x},P_{y}\right)=\left(1/2,0\right)$ for $\kappa_{1}<\kappa_{2}$. A pair of edge states appear under the OBC in the x direction [Fig. 4(a1)]. The arrow at certain $k_{y}$ counterclockwise rotates once as $k_{x}$ varies an entire period from −π to π [Fig. 4(a2)], which corresponds to $P_{x}=1/2$. The edge states $E_{L}$ and $E_{R}$ appear on the left and right boundaries [Fig. 4(a3)] and copropagate along the vertical direction. The polarization in the purple regions is $\left(P_{x},P_{y}\right)=\left(0,1/2\right)$, a pair of edge states appear under the OBC in the y direction [Fig. 4(b1)]. The arrow at certain $k_{x}$ counterclockwise rotates once as $k_{y}$ varies an entire period from −π to π [Fig. 4(b2)], which corresponds to $P_{y}=1/2$. The edge states $E_{T}$ and $E_{B}$ appear on the top and bottom boundaries [Fig. 4(b3)] and copropagate along the horizontal direction. As illustrated in Fig. 4(a3) and (b3), the edge states localized on the left and bottom (right and top) boundaries are mostly localized on the sublattice A (B). The polarization in the green regions is $\left(P_{x},P_{y}\right)=\left(1/2,-1/2\right)$. A pair of edge states appear under the OBC in the x- and y-direction, respectively [Fig. 4(c1)]. The arrow at certain $k_{y}$ ($k_{x}$) counterclockwise (clockwise) rotates once as $k_{x}$ ($k_{y}$) varies an entire period from −π to π [Fig. 4(c2)], which corresponds to $P_{x}=1/2$ ($P_{y}=-1/2$). The edge state localized on the left (right) boundary is still localized on the sublattice A (B), but the edge state localized on the bottom (top) boundary changes to be mostly localized on the sublattice B (A) [Fig. 4(c3)]. Topological phases with $P_{y}=1/2$ satisfy $\left|w_{1}\right|>\left|w_{2}\right|$ and topological phases with $P_{y}=-1/2$ satisfy $\left|w_{1}\right|<\left|w_{2}\right|$, then, the edge states $E_{T}$ and $E_{B}$ mainly occupy different sublattices (see Supplementary C). The square lattice lacks $C_{4}$ symmetry protection, thus $P_{x}\cdot P_{y}\neq 0$ is a first-order topological phase [22]. The edge states present on the four boundaries and copropagate along both the vertical and horizontal directions. Although the four boundaries are bidirectional, the edge states *cannot* clockwise or counterclockwise circulate along the boundaries similar to two pairs of opposite chiral edge states. In addition, $P_{x}\cdot P_{y}\neq 0$ (=0) does not indicate the presence (absence) of corner states [23], [24], [25] (see Supplementary D).

Notably, the robust propagation dynamics associated with the edge states are important in practical applications. The robustness to obstacles is a consequence of the unidirectionality from the time-reversal symmetry breaking, where the counterpropagating channel is closed and the backscattering is completely suppressed. This is a typical feature of the chiral edge states. In contrast to the unidirectional wave propagation of chiral edge states, we emphasize that the bidirectional wave propagation of the synchronized in-gap edge states cannot robustly pass through the intentionally embedded obstacles due to the presence of backscattering channel as a consequence of the time-reversal symmetry. Nevertheless, the propagation dynamics of the in-gap edge states are still robust to random disorder. The robustness originates from the band gap protection. In practice, the use of edge states for topological transport seldom encounters obstacles, and the propagations along the boundaries are rarely affected by the random fabrication errors and environmental perturbations without the emergence of obvious backscattering. Thus, the robustness of in-gap edge states protected by the wide band gap is enough for the practical applications. The robustness of edge states to certain disorder closely depends on whether the disorder breaks the symmetry of the topological system. The degeneracy of in-gap edge states is destroyed when the inversion symmetry is absent in the presence of random disorder. Although the edge states are slightly affected by the disorder with a splitting in the energy, the band gap is still able to provide the topological protection for their propagation dynamics.

**Fig. 5:**
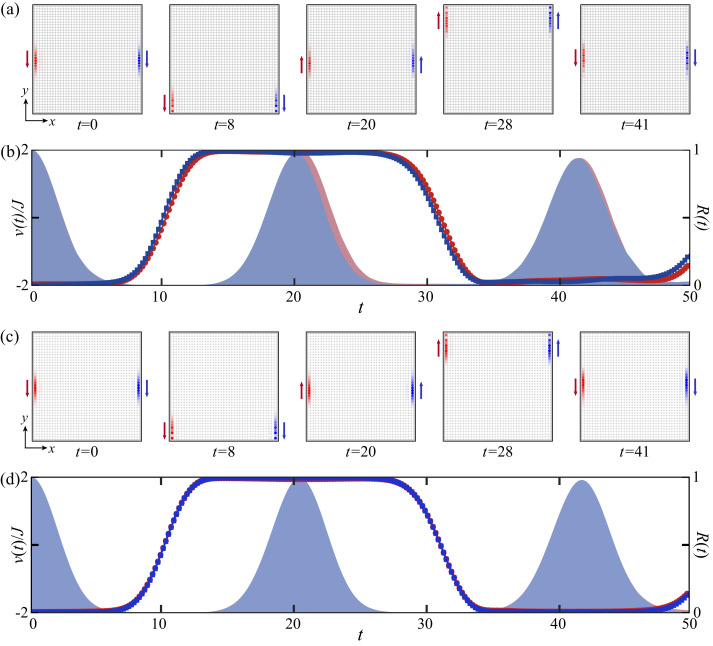
(a) Snapshots of copropagation along the vertical boundaries and (b) the propagation velocity and return probability for the couplings randomly deviated from the set strengths within the range of [−5%,5%]. (c) Snapshots of copropagation along the vertical boundaries and (d) the propagation velocity and return probability for the detunings randomly deviated within the range of [−5%,5%] of the unit coupling J. The initial excitation is the Gaussian wave packet of the edge state with the momentum $k_{y}=\pi /2$. Red and blue lines indicate the propagation velocities ν(t) of the wave packets on the left and right boundaries, respectively. Red and blue areas indicate the return probabilities R(t) of the wave packets on the left and right boundaries, respectively. The lattice size is 40×40, the system parameters are from Fig. 4(a). The unit of time is $J^{-1}$.

To verify our theoretical analysis, we use the Gaussian wave packet to simulate the propagation dynamics [15], [17], [26], [27], [28]. Figure 5 numerically demonstrates the robust copropagation of Gaussian wave packet of the edge state excitation from the topological phase $\left(P_{x},P_{y}\right)=\left(1/2,0\right)$ in the presence of random disorder. The left (right) edge state $E_{L}$ ($E_{R}$) is exactly localized on the sublattice A (B). The edge state energy is $E_{L,R}\left(k_{y}\right)=2J\cos k_{y}$. The velocity of edge state excitation is $\nu_{L,R}\left(k_{y}\right)=dE_{L,R}\left(k_{y}\right)/dk_{y}$. In the numerical simulations, the initial excitation |Ψ(0)〉 is a Gaussian wave packet of the edge state localized on the left/right boundary

(7) $$|\Psi (0)\rangle =\Omega \sum_{k_{y}}e^{-{\left(k_{y}-k_{0}\right)}^{2}/\left(2\alpha^{2}\right)}e^{-iN_{c}\left(k_{y}-k_{0}\right)}e^{ik_{y}n}|\psi_{L/R}\rangle ,$$

where $|\psi_{L/R}\rangle$ is the wave function of the left/right edge state in the momentum space (see Supplementary C) and n is the index of unit cell. $k_{0}$ is the central momentum, $N_{c}$ is the center, and Ω is the normalization coefficient of the Gaussian wave packet. The parameter α controls the width of the Gaussian wave packet. A Gaussian wave packet in momentum space under the Fourier transformation remains a Gaussian wave packet in real space as shown in Fig. 5. |Ψ(t)〉 is the time evolution of the initial excitation |Ψ(0)〉 in the square lattice under OBCs on both directions at the moment t. The return probability is $R\left(t\right)=\sum_{j}{\left|\Psi_{j}\left(0\right)\Psi_{j}\left(t\right)\right|}^{2}$, where the subscript j is the site number. The dynamics of the Gaussian wave packet excitation reflects the dispersion of the edge state at the momentum $k_{0}$ in the band structure under open boundary condition of the square lattice as shown in Fig. 4. The square lattice size is 40×40, the Gaussian wave packet returns to its initial position with an opposite velocity at about the moment $t=20J^{-1}$ for the first time. Thus, the velocity of the edge state excitation along the boundary in the numerical simulation is about 2J. This is consistent with the propagation velocity $\nu_{L,R}\left(k_{0}\right)$ obtained in our theoretical analysis. In addition, the edge state excitations with opposite momenta are degenerate, and they propagate in opposite directions. However, the degenerate edge state excitations in their propagations do not merge with each other or backward propagate until being reflected back at the corner of the square lattice.

The edge state excitations on both the left and right boundaries at the central momentum $k_{y}=\pi /2$ in the presence of random coupling disorder within the range of [−5%,5%] are performed in Figs. 5(a) and (b). Figure 5(a) provides the snapshots for the synchronized copropagation. Figure 5(b) exhibits the velocity and return probability during the copropagation. The coupling disorder does not obviously affect the robust copropagation of the edge states even if the deviation from the exact values of couplings reaches the range of [−10%,10%] (see Supplementary E). The random detuning in the resonator frequency is the on-site Anderson disorder of the time-reversal symmetric square lattice. Although the Anderson disorder may solely create nontrivial band topology known as the topological Anderson insulator [29], [30], [31], the random detunings in the time-reversal symmetric square lattice break the inversion symmetry of the square lattice and slightly disturb the edge states. Nevertheless, the copropagation is robust to detuning disorder under the band gap protection. The edge state excitations on both the left and right boundaries at the central momentum $k_{y}=\pi /2$ in the presence of random detuning disorder are performed in Figs. 5(c) and (d). Figure 5(c) provides the snapshots for the synchronized copropagation. Figure 5(d) exhibits the velocity and return probability during the copropagation. In the numerical simulations, the Gaussian wave packets copropagate along the boundaries, and bounce back and forth at the corners without obvious backscattering in the copropagation for weak disorder. The Gaussian wave packets complete a cyclic process after returning to the initial position with the same velocity. The disorder-immune robust copropagation dynamics of the edge state excitation verifies the theoretical prediction on the robustness.

In addition, the bidirectionality of the in-gap edge states protected by the time-reversal symmetry naturally provides a possibility for picking the propagation direction along the boundaries in a desirable manner via selectively choosing the momenta of edge state excitations. This offers a topological insulator to simultaneously support robust copropagation and counterpropagation. The disorder-immune robust counterpropagation is realized by selectively exciting the in-gap edge states with the momentum $k_{y}=\pi /2$ on the left boundary and the momentum $k_{y}=-\pi /2$ on the right boundary (see Supplementary F).

## 3 Conclusion

In conclusion, we have proposed a novel time-reversal symmetric topological insulator supporting the synchronized in-gap edge states. The associated copropagation dynamics avoids the participation of bulk states and prevents the edge state excitations from being scattered into the bulk. The discovery of synchronized in-gap edge states in the time-reversal symmetric topological insulators has expanded our understanding of two-dimensional topological phases of matter, enriched the band topology, and provided new opportunities for topological light steering. The robust copropagation can be generalized to higher-dimension [32], [33], [34] and higher-order topology [35]. It is interesting to further explore robust copropagation in nonlinear [36] and non-Hermitian [37], [38], [39] photonic systems. In addition, the robust copropagation along the parallel boundaries provides versatile ways of wave transport for robust light steering [40]. The unique topology in the time-reversal symmetric square lattice enriched by the anisotropic long-range couplings is highlighted. It is also convenient to realize topological lattices without magnetic flux in the acoustic [41], [42], [43], [44], [45], [46], [47], [48], electronic [49], and mechanical [50] systems. Our findings pave the way for future explorations on the fundamentals and applications of time-reversal symmetric topological metamaterials.

## Declaration of competing interest

The authors declare that they have no conflicts of interest in this work.

## Appendix A Supplementary materials

Supplementary Data S1 Supplementary A provides the polarization for topological characterization. Supplementary B provides the edge states in the topological metallic phase. Supplementary C provides the energies and wave functions of edge states. Supplementary D provides the difference between the proposed square lattice and the two-dimensional Su-Schrieffer-Heeger model. Supplementary E provides the robust copropagation of in-gap edge states in the presence of random coupling disorder within the range of [−10%,10%]. Supplementary F provides the robust counterpropagation of in-gap edge states for the excitation on the parallel boundaries with the opposite momenta. Supplementary Raw Research Data. This is open data under the CC BY license http://creativecommons.org/licenses/by/4.0/ Supplementary Data S1
