# Supplementary material for: Synchronized in-gap edge states and robust copropagation in topological insulators without magnetic flux
Source: Fundam Res. 2025 Jan 21;6(4):2153–9. doi: 10.1016/j.fmre.2025.01.005 (PMC13424711; doi:10.1016/j.fmre.2025.01.005)
Supplement: Supplementary Data S1 — Supplementary A provides the polarization for topological characterization. Supplementary B provides the edge states in the topological metallic phase. Supplementary C provides the energies and wave functions of edge states. Supplementary D provides the difference between the proposed square lattice and the two-dimensional Su-Schrieffer-Heeger model. Supplementary E provides the robust copropagation of in-gap edge states in the presence of random coupling disorder within the range of [−10%,10%]. Supplementary F provides the robust counterpropagation of in-gap edge states for the excitation on the parallel boundaries with the opposite momenta. Supplementary Raw Research Data. This is open data under the CC BY license http://creativecommons.org/licenses/by/4.0/ [file mmc1.pdf]

Supplementary Materials for "Synchronized in-gap edge states and robust copropagation in topological insulators without magnetic flux"

Liangcai Xie, Tianyi He, and Liang Jin*
School of Physics, Nankai University, Tianjin 300071, China

A. The polarization for topological characterization

The polarization of the square lattice in Fig. 3(a) of the main text is visualized in Fig. 4(a2), Fig. 4(b2), and Fig. 4(c2) of the main text. In the following, we prove the validity in detail. The eigenstate of the Bloch Hamiltonian $h(\mathbf{k}) = \mathbf{d}(\mathbf{k}) \cdot \sigma + d_0(\mathbf{k})\sigma_0$ can be written in the form of

$$|\psi_\pm(\mathbf{k})\rangle = \frac{1}{\sqrt{2}}\begin{pmatrix} \pm 1 \\ e^{i\varphi(\mathbf{k})} \end{pmatrix}, e^{i\varphi(\mathbf{k})} = \frac{d_x + i d_y}{|\mathbf{d}|}. \tag{1}$$

The $d_0(\mathbf{k})\sigma_0$ term in the Bloch Hamiltonian does not alter the eigenstate. The polarization defined by the eigenstate of $h(\mathbf{k})$ is identical to the polarization defined by the eigenstate of $\mathbf{d}(\mathbf{k}) \cdot \sigma$. The effective magnetic field term $\mathbf{d}(\mathbf{k}) \cdot \sigma = d_x\sigma_x + d_y\sigma_y$ of the square lattice has the chiral symmetry. The polarization of a system with chiral symmetry is associated with the winding of effective magnetic field.

The components of the wave polarization projected in the x and y directions are defined as

$$P_{\pm,x} = \frac{1}{(2\pi)^2}\int_{-\pi}^{\pi}\int_{-\pi}^{\pi} -i\langle\psi_\pm(k_x,k_y)|\partial_{k_x}\psi_\pm(k_x,k_y)\rangle\, dk_x dk_y \tag{2}$$

$$P_{\pm,y} = \frac{1}{(2\pi)^2}\int_{-\pi}^{\pi}\int_{-\pi}^{\pi} -i\langle\psi_\pm(k_x,k_y)|\partial_{k_y}\psi_\pm(k_x,k_y)\rangle\, dk_x dk_y \tag{3}$$

The Zak phase at fixed k_y is defined as $Z_{\pm,x}(k_y) = \int_{-\pi}^{\pi} -i\langle\psi_\pm(k_x,k_y)|\partial_{k_x}\psi_\pm(k_x,k_y)\rangle\, dk_x$. From the eigenstate in Eq. (1), we obtain $Z_{\pm,x}(k_y) = \int_{-\pi}^{\pi} d\varphi(\mathbf{k})/2$. Then, we obtain the polarization in the form of Zak phase as $P_{\pm,x} = (2\pi)^{-2}\int_{-\pi}^{\pi} Z_{\pm,x}(k_y) dk_y$, i.e.,

$$P_{\pm,x} = \frac{1}{8\pi^2}\int_{-\pi}^{\pi} d\varphi(\mathbf{k})\int_{-\pi}^{\pi} dk_y. \tag{4}$$

The integral of the argument $\varphi(\mathbf{k})$ is associated with the winding of the effective magnetic field $\mathbf{d}(\mathbf{k})$. In the nontrivial phase with the in-gap edge state, $\varphi(\mathbf{k})$ varies $\pm 2\pi$ as k_x varying an entire period from $-\pi$ to π for every fixed k_y. Thus, the Zak phase is $\pm\pi$ for every fixed k_y and $P_{\pm,x} = \pm 1/2$. We observe in Fig. 4(a2) of the main text that for any k_y, the effective magnetic field marked by the arrows rotates once as k_x varying an entire period from $-\pi$ to π. The polarization in the y direction has similar result as visualized in Fig. 4(b2).

B. The absence of edge states in the inseparable region of topological metallic phase

The symmetry protected quantized polarization captures topologically in-gap edge states. In the square lattice, although the chiral symmetry breaks, the inversion symmetry is preserved and results in the quantized polarization. The chiral symmetry breaking arises from the emergence of the identical matrix $d_0(\mathbf{k}) = 2J\cos k_y$ in the Bloch Hamiltonian, which can also alter the system from a topological insulator to a topological metal. The topological metal does not have a full band gap. The two energy bands of the one-dimensional projection of topological metal may partially cover each other. Then, the edge states predicted by the nonzero polarization disappear within the inseparable region of the energy band.

Since $d_0(\mathbf{k}) = 2J\cos k_y$ is a function of the momentum k_y, thus, the one-dimensional projection energy band in the y-direction is always separable. The in-gap edge states $E_{L,R}(k_y)$ predicted by the bulk polarization P_x are unaffected. The absence of edge states in the inseparable region of the topological metallic phase only occurs for the edge states $E_{B,T}(k_x)$ in the other direction.

The edge states are typically characterized by their localization, and in the next section we analytically solve the edge states in both directions. We take the left and right edge states as an example to determine the presence of in-gap edge states. For the left and right edge states $E_{L,R}(k_y)$, if the decay factor $|\rho_{L,R}| < 1$ is satisfied for the momentum

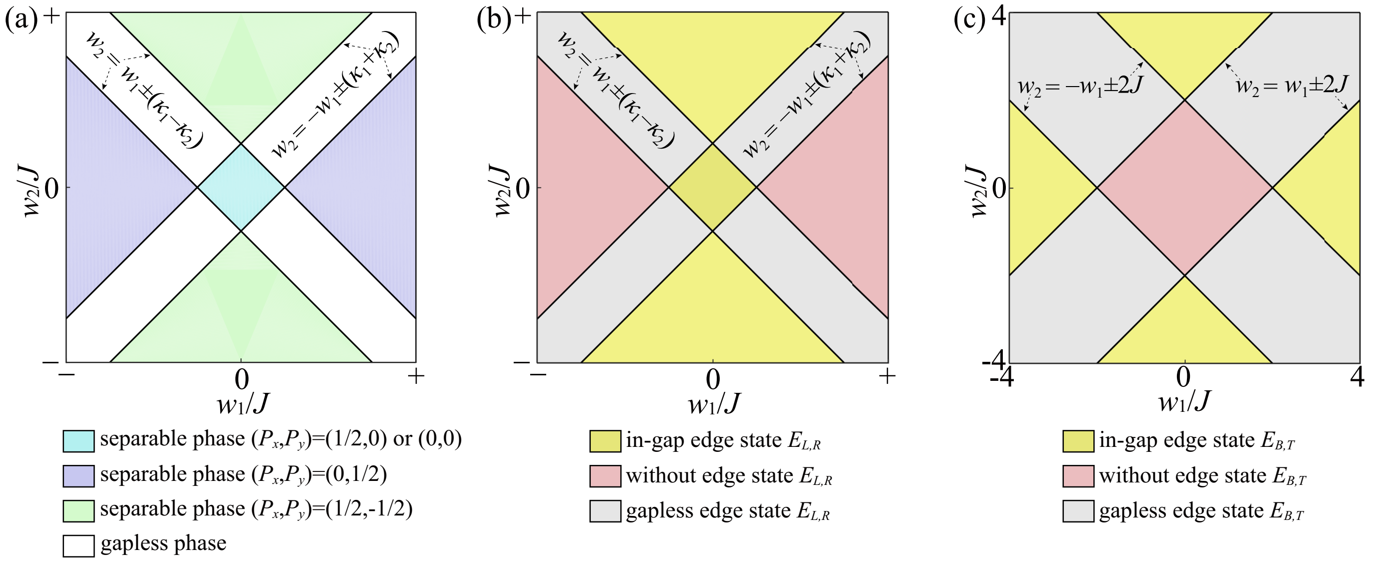

Supplemental Figure 1. (a) Polarization distribution. (b) In-gap state distributions under the OBC in the x-direction. (c) In-gap state distributions under the OBC in the y-direction. (a) and (b) are dependent of κ_1 and κ_2. (c) is independent of κ_1 and κ_2. Without the energy band overlap, the nonzero polarization characterizes the presence of edge states.

$k_y \in [-\pi, \pi]$, the left and right edge states must be in-gap throughout the Brillouin zone; if the decay factor $|\rho_{L,R}| < 1$ is satisfied only for part of the momentum $k_y \in [-\pi, \pi]$, then the edge states are truncated and there is an overlap of the projected energy bands; if the decay factor $|\rho_{L,R}| < 1$ is not satisfied for any momentum $k_y \in [-\pi, \pi]$, then the edge states are absent. The results obtained from this analysis are consistent with the topological phase diagram, which is shown in Supplemental Figures 1(a) and 1(b). That is, the boundary determining the in-gap edge state $E_{L,R}(k_y)$ is the band gap closing boundary $w_2 = w_1 \pm (\kappa_1 - \kappa_2)$ and $w_2 = -w_1 \pm (\kappa_1 + \kappa_2)$.

The distribution boundaries for the bottom and top states $E_{B,T}(k_x)$ are $w_2 = \pm w_1 \pm 2J$ as shown in Supplemental Figure 1(c). The yellow regions indicate the presence of in-gap edge states without energy overlap in the projection bands, the gray regions indicate the presence of gapless edge states with energy overlap in the projection bands, and the red regions indicate the absence of in-gap edge states. Obviously, these boundaries are different from the boundaries of topological phase transition of Supplemental Figure 1(a). By combining these two kinds of boundaries, we completely capture the in-gap edge states. If $|\kappa_1 + \kappa_2| \geq 2J$ and $|\kappa_1 - \kappa_2| \geq 2J$, the energy overlap is absent in the projection bands and the in-gap edge states $E_{B,T}(k_x)$ predicted by P_y are not affected. The energy overlap is present in the projection bands for all the other cases. For example, when $\kappa_1 = J$, $\kappa_2 = 4J$, the projection bands do not have energy band overlap, and the in-gap edge states are present in both the green and purple regions of Fig. 3(c) of the main text. In this case, the bulk polarization P_y accurately captures the in-gap edge states and the bulk-boundary correspondence is valid in the entire Brillouin zone.

Supplemental Figure 2 demonstrates a typical example with energy overlap in the projection bands. When $\kappa_1 = J$, $\kappa_2 = J$, the topological phase diagram without considering energy band overlap is shown in Supplemental Figure 2(a). From the boundaries of the edge state distribution, the energy band overlap is present in the projection bands, and the in-gap edge states disappear in the gray regions as compared in Supplemental Figure 2(b), i.e., the in-gap edge states predicted by P_y are destroyed by the energy overlap. The purple regions do not have energy overlap in the projection bands, and the in-gap edge states predicted by P_y are not affected. The dashed black line $w_2 = \pm w_1 \pm 2J$ divides the region with and without energy band overlap. The white regions are the gapless phase with inseparable energy bands.

Supplemental Figures 2(c)-2(f) demonstrate the energy band overlap under two parameters as indicated by the red dots in Supplemental Figure 2(b). Supplemental Figure 2(c) illustrates the critical case that the projected energy bands under open boundary condition in the y-direction are touched. The in-gap edge states $E_{B(T)}$ and the bulk bands intersect at $k_x = \pi$. Supplemental Figure 2(d) illustrates the case that the projected bands under open boundary condition in the y-direction have overlap. The in-gap edge states $E_{B(T)}$ are partially dissolved into the bulk bands, as evidenced from the inverse participation ratio shown in Supplemental Figure 2(f). Notably, the in-gap edge state is completely dissolved into the bulk in certain cases of the gray regions.

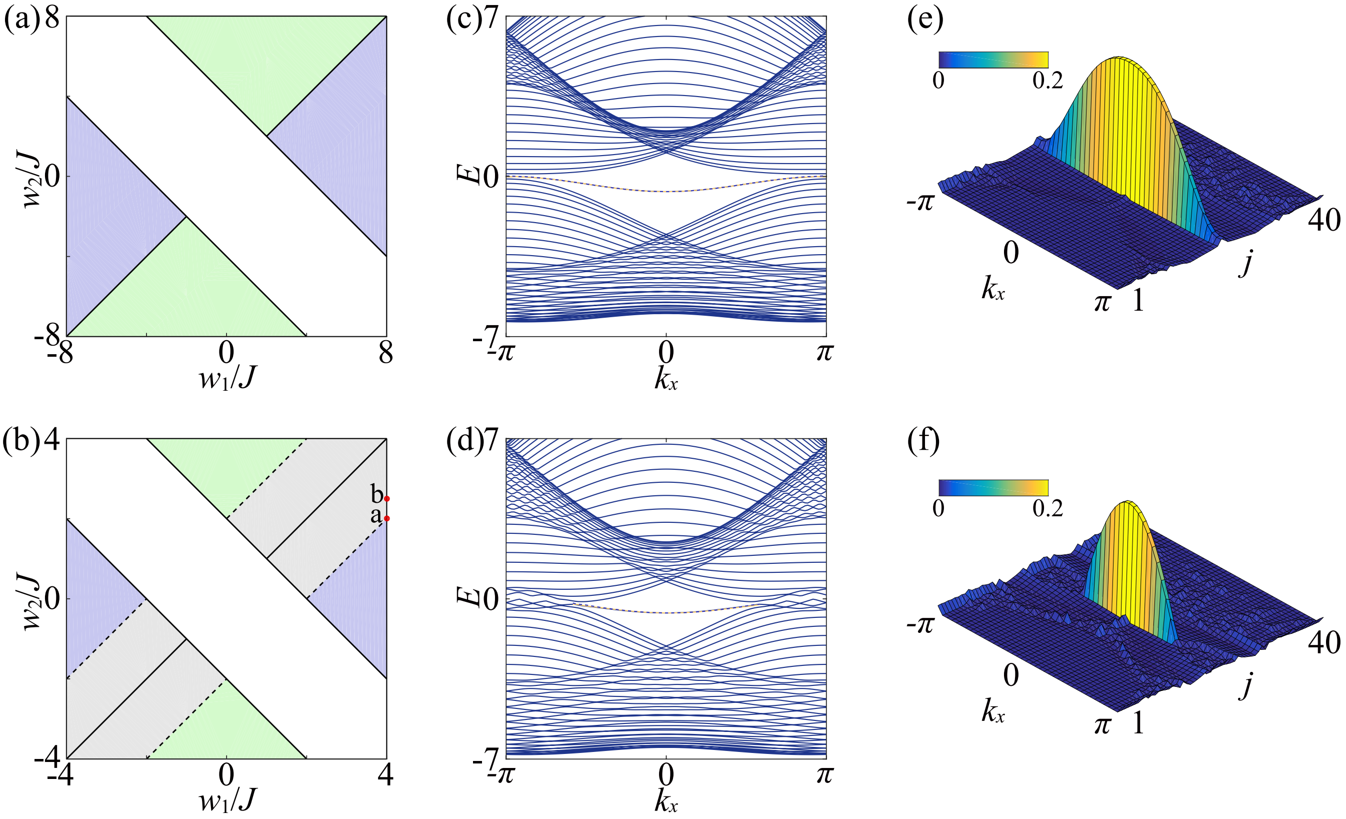

Supplemental Figure 2. (a) Topological phase diagrams not considering band overlap. (b) Topological phase diagrams with band overlap. The parameters are set to $\kappa_1 = \kappa_2 = J$ for (a) and (b). (c) and (e) In-gap edge states with no overlap and inverse participation ratio for all states. (d) and (f) Gapless edge states with overlap and inverse participation ratio for all states. The parameters are set to $w_1 = 4J$, and $w_2 = 2J$ in (c) and (e) as indicated by the red dot a in (b) and $w_2 = 2.5J$ in (d) and (f) as indicated by the red dot b in (b).

C. The edge states of square lattice

The edge states with energies $E_{L,R}(k_y)$ exist under the open boundary condition in the x direction, and the edge states with energies $E_{B,T}(k_x)$ exist under the open boundary condition in the y direction. The wave functions for the edge states are expressed as $|\psi_{L,R,B,T}\rangle = (\varphi_{1,A}, \varphi_{1,B}, \cdots, \varphi_{n,A}, \varphi_{n,B}, \cdots, \varphi_{N,A}, \varphi_{N,B})$, where N is the total number of unit cells along the direction with the open boundary condition and n indexes the unit cell. $\varphi_{n,A}$ ($\varphi_{n,B}$) represents the component of wave function on the sublattice A (B) in the n-th unit cell. The wave functions of the four edge states are analytically obtained at the infinite-size limitation $N \to \infty$.

Applying the Fourier transformation in the y direction, we obtain the one-dimensional projection lattice as schematically illustrated in Supplemental Figure 3(a). The one-dimensional projection lattice is a Rice-Mele chain with a k_y-dependent uniform coupling along the x direction and the sites of sublattices A and B alternately appear. The wave functions $|\psi_L\rangle$ and $|\psi_R\rangle$ are for the edge states with energies $E_L(k_y)$ and $E_R(k_y)$

$$E_{L,R}(k_y) = 2J\cos k_y \tag{5}$$

For the edge state $|\psi_L\rangle$, the component of $|\psi_L\rangle$ at the sublattice B vanishes ($\varphi_{n,B} = 0$). For the edge state $|\psi_R\rangle$, the component of $|\psi_R\rangle$ at the sublattice A vanishes ($\varphi_{n,A} = 0$). The wave functions are expressed as

$$|\psi_L\rangle = (1, 0, \cdots, \rho_L^{n-1}, 0, \cdots, \rho_L^{N-1}, 0), |\psi_R\rangle = (0, \rho_R^{N-1}, \cdots, 0, \rho_R^{N-n}, \cdots, 0, 1), \tag{6}$$

where the decay factors are $\rho_L = -\left(\kappa_1 + w_1 e^{ik_y}\right) / \left(\kappa_2 + w_2 e^{-ik_y}\right)$ and $\rho_R = \rho_L^*$. As shown in Fig. 4 of the main text, the edge state $|\psi_L\rangle$ only localizes on the sublattice A and decays exponentially from left to right of the square

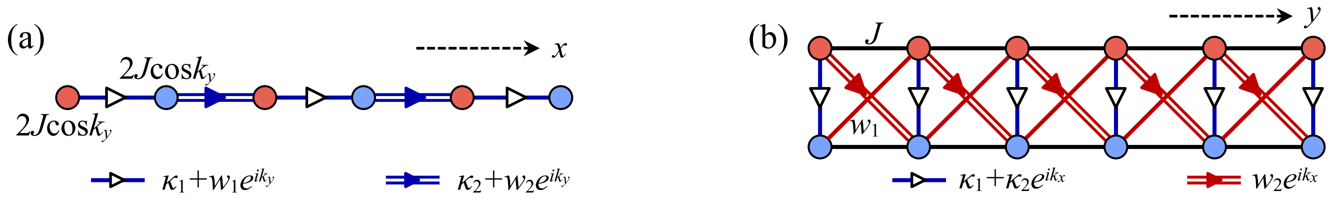

Supplemental Figure 3. Schematic of the 1D projection lattice for the 2D square lattice. The (a) Rice-Mele chain and (b) Creutz ladder are obtained through applying the Fourier transformation in the y direction and in the x direction, respectively.

lattice; and the edge state $|\psi_R\rangle$ only localizes on the sublattice B and decays exponentially from right to left of the square lattice.

Applying the Fourier transformation in the x direction, we obtain the one-dimensional projection lattice as schematically illustrated in Supplemental Figure 3(b). The one-dimensional projection lattice is a Creutz ladder with the two legs along the y direction, one ladder-leg site belongs to the sublattice A and the other ladder-leg site belongs to the sublattice B. The ladder rung coupling is k_x-dependent. The wave functions $|\psi_B\rangle$ and $|\psi_T\rangle$ are for the edge states with energies $E_B(k_x)$ and $E_T(k_x)$. Solving the Hamiltonian, we observe that the energy of edge state is determined from the following expression

$$\left(\beta w + w_2 e^{-ik_x}\right)\left(E - \left(\kappa_1 + \kappa_2 e^{ik_x}\right)\beta w\right) = \left(w_1 \beta w + 1\right)\left(E\beta w - \left(\kappa_1 + \kappa_2 e^{-ik_x}\right)\right). \tag{7}$$

where $w = w_1 - w_2 e^{-ik_x}$. The Hermiticity of the system results in $E_{B,T}(k_x) = Re(E)$ and β is determined from $Im(E) = 0$. We obtain β in the form of

$$\beta = \frac{-w_1^2 + w_2^2 - \sqrt{\left(w_1^2 - w_2^2\right)^2 - 4J^2\left(w_1^2 + w_2^2\right) + 8J^2 w_1 w_2 \cos k_x}}{2J\left(w_1^2 + w_2^2 - 2w_1 w_2 \cos k_x\right)}. \tag{8}$$

It is noted that β is independent of κ_1 and κ_2. For the edge state $|\psi_B\rangle$, if we take $\varphi_{1,B} = 1$, the wave function of bottom state is expressed

$$\begin{cases} \varphi_{2,B} = \frac{E\beta w - \left(\kappa_1 + \kappa_2 e^{-ik_x}\right)}{J\beta w + w_2 e^{-ik_x}} \varphi_{1,B}, \\ \varphi_{n,A} = \beta w \varphi_{n,B}, \\ \varphi_{n,B} = \frac{E\beta w - \left(\kappa_1 + \kappa_2 e^{-ik_x}\right)}{J\beta w + w_2 e^{-ik_x}} \varphi_{n-1,B} - \frac{J\beta w + w_1}{J\beta w + w_2 e^{-ik_x}} \varphi_{n-2,B}, (3 \le n \le N). \end{cases} \tag{9}$$

For the edge state $|\psi_T\rangle$, if we take $\varphi_{N,A} = 1$, the wave function of top state is expressed as

$$\begin{cases} \varphi_{N-1,A} = \frac{E\beta w^* - \left(\kappa_1 + \kappa_2 e^{ik_x}\right)}{J\beta w^* + w_2 e^{ik_x}} \varphi_{N,A}, \\ \varphi_{N-n+1,B} = \beta w^* \varphi_{N-n+1,A}, \\ \varphi_{N-n+1,A} = \frac{E\beta w^* - \left(\kappa_1 + \kappa_2 e^{ik_x}\right)}{J\beta w^* + w_2 e^{ik_x}} \varphi_{N-n+2,A} - \frac{J\beta w^* + w_1}{J\beta w + w_2 e^{ik_x}} \varphi_{N-n+3,A}, (3 \le n \le N). \end{cases} \tag{10}$$

For topological nontrivial phases of $P_y = 1/2$ with $|w_1| > |w_2|$, the solution of β satisfies $|\beta w| > 1$. Consequently, the edge states $|\psi_B\rangle$ and $|\psi_T\rangle$ are predominantly distributed on the sublattices A and B, respectively. Conversely, for topological nontrivial phases of $P_y = -1/2$ with $|w_1| < |w_2|$, the situation is reversed, with the edge states $|\psi_B\rangle$ and $|\psi_T\rangle$ predominantly distributed on the sublattices B and A, respectively. The edge state $|\psi_B\rangle$ decays exponentially from bottom to top of the square lattice, while the edge state $|\psi_T\rangle$ decays exponentially from top to bottom of the square lattice as illustrated in Fig. 4 of the main text.

D. Different topological phases of the square lattice model and two-dimensional Su-Schrieffer-Heeger model

We emphasize that the square lattice model supporting the first-order topological phase is intrinsically different from these two-dimensional SSH models supporting the higher-order topological phase in terms of symmetry, topological classification, edge states, and corner states. (i) The chiral symmetry is broken in the square lattice model, whereas it preserves in the two-dimensional SSH model. Then, the former belongs to the AI class, whereas the latter belongs to

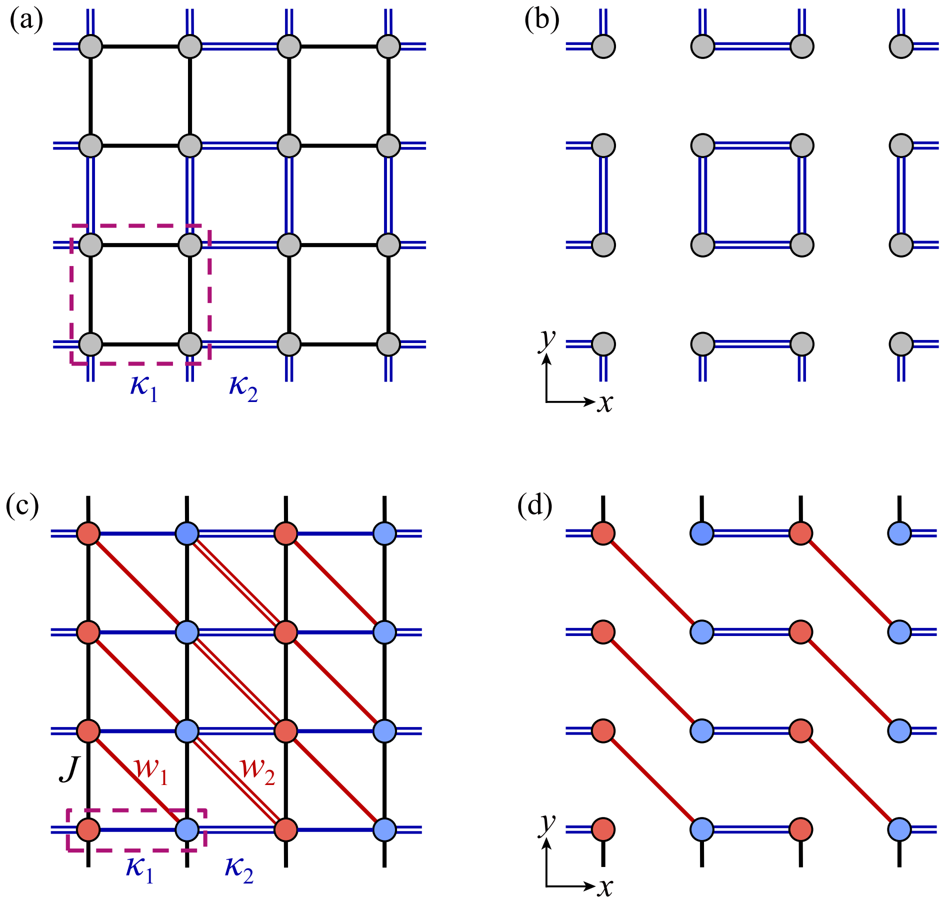

Supplemental Figure 4. (a) Schematic of the two-dimensional SSH model. (b) Four corner states of (a) at $\kappa_1 = 0$, $\kappa_2 \neq 0$. (c) Schematic of the square lattice model. (d) Two corner modes appear at the lower-left and upper-right corners of (c) at $\kappa_1 = 0$, $\kappa_2 \neq 0$, $w_1 \neq 0$, $w_2 = 0$, $J = 0$.

the BDI class in the tenfold-way topological classification. (ii) The edge states of the square lattice model are a pair of edge states in each direction, whereas the edge states of the two-dimensional SSH model are two pairs of edge states as a consequence that the former is a two-band model and the latter is a four-band model. (iii) The quantized bulk polarization $P_x \cdot P_y \neq 0$ $(= 0)$ in the two-dimensional SSH model indicates the presence (absence) of corner states, whereas this is not necessarily valid in the square lattice model.

The two-dimensional Su-Schrieffer-Heeger (SSH) model [Supplemental Figure 4(a)] generally supports two types of higher-order topological phases with topological corner states, one type with quantized quadrupole moment and the other type without quadrupole moment. The former case is the Benalcazar-Bernevig-Hughes (BBH) model, which is a two-dimensional SSH model with π flux in each plaquette and the two-fold degenerate energy bands [1]. The quadrupole moments are associated with edge polarizations and are protected by C_4 symmetry, which guarantees the quantized corner charge at each corner, manifesting as the existence of four degenerate corner states respectively localized at the four corners [Supplemental Figure 4(b)]. The latter case is a two-dimensional SSH model without any flux in each plaquette and the single occupied energy band. The quadrupole moment is absent. The edge polarizations reduce to the bulk polarizations and the existence of corner state is determined by the nonvanishing values of $P_x \cdot P_y$ [2]. Then, four corner states appear only if both P_x and P_y are nonzero. The two-dimensional SSH model also supports copropagation in the higher-order topological phase with $(P_x, P_y) = (1/2, 1/2)$. The spectrum for the two-dimensional SSH model under open boundary condition exhibits two pairs of in-gap edge states in both directions. The edge state excitations within the band gap copropagate along the boundaries and are reflected back at the corners because of the mismatch of the on-resonant state in the other direction.

The square lattice model [Supplemental Figure 4(c)] has the topological phase $(P_x, P_y) = (1/2, -1/2)$. Since the C_2 symmetry of the system is preserved while the C_4 symmetry is broken, the polarization is quantized, but the corner states are not topologically protected. The corner states still exist even if $P_x \cdot P_y = 0$. For instance, at

$J = \kappa_1 = w_2 = 0$, if $\kappa_2 < w_1$, the polarizations are $P_x = 0$ and $P_y = 1/2$. Under the open boundary conditions in both horizontal and vertical directions, the single sites at the lower-left and upper-right corners are decoupled from the other sites, forming two corner states [Supplemental Figure 4(d)]. In addition, the nearest-neighbor coupling J may also affect the existence of corner states.

E. Copropagation of in-gap edge states in the square lattice

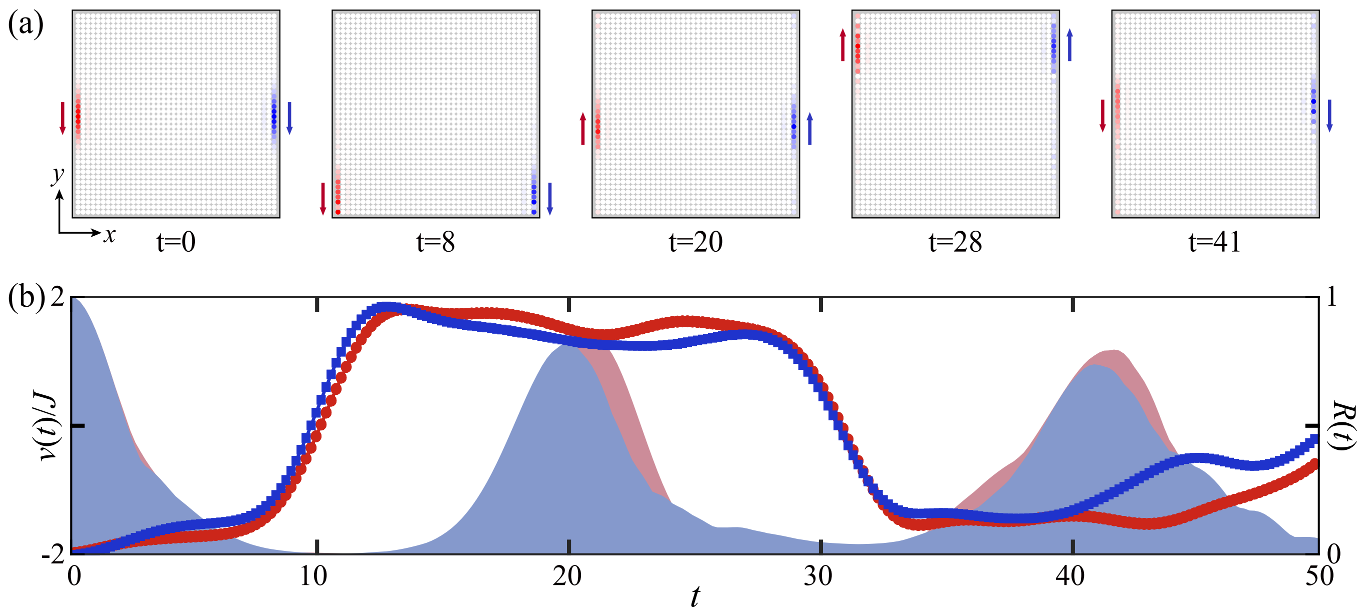

Supplemental Figure 5. (a) Snapshots of copropagation along the vertical boundaries and (b) the propagation velocity and return probability for the couplings randomly deviated from the set strengths within the range of $[-10\%, 10\%]$. The Gaussian wave packet of the initial edge state excitation on both the left and right boundaries has the momentum $k_y = \pi/2$. Red and blue lines indicate the propagation velocities $\nu(t)$ of the wave packets on the left and right boundaries, respectively. Red and blue areas indicate the return probabilities $R(t)$ of the wave packets on the left and right boundaries, respectively. The lattice size is 40×40, the system parameters are from Fig. 4(a). The unit of time is J^{-1}.

In the topological insulating phase of the time-reversal symmetric square lattice, the bulk states are completely off-resonant with the in-gap edge states. Thus, the edge state excitations propagating along the boundaries are reflected back toward the opposite direction at the corners. Similarly, the large obstacles laid on the boundaries of the square lattice may cause backscattering of the edge state excitations. The presence of backscattering channels is the reason for the fact that the disorder in principle must not be too large. However, the presence of backscattering channels does not mean that the dynamics of edge state excitations along the boundaries are not robust to disorder. The band gap can protect the robust propagation along the boundaries.

In Fig. 5 of the main text, we show the robust copropagation of the synchronized in-gap edge states for the weak disorder within the range of $[-5\%, 5\%]$. Supplemental Figure 5 shows the robust copropagation of the synchronized in-gap edge state for the moderate disorder within the range of $[-10\%, 10\%]$. From the numerical simulation, we notice that the disorder does not obviously affect the robust copropagations of the synchronized in-gap edge states even if the deviation from the exact values of the couplings reaches the range of $[-10\%, 10\%]$. The backscattering is observed in the copropagation of the synchronized in-gap edge state for the large disorder within the range of $[-15\%, 15\%]$.

F. Counterpropagation of in-gap edge states in the square lattice

The bidirectionality is a key feature of the in-gap edge states in the time-reversal symmetric topological insulators. Consequently, this naturally provides a possibility for picking the propagation direction along the boundaries in a desirable manner through selectively exciting the edge states. The copropagation of in-gap edge states along the parallel boundaries is realized when the excitation of in-gap edge states on the parallel boundaries has the same

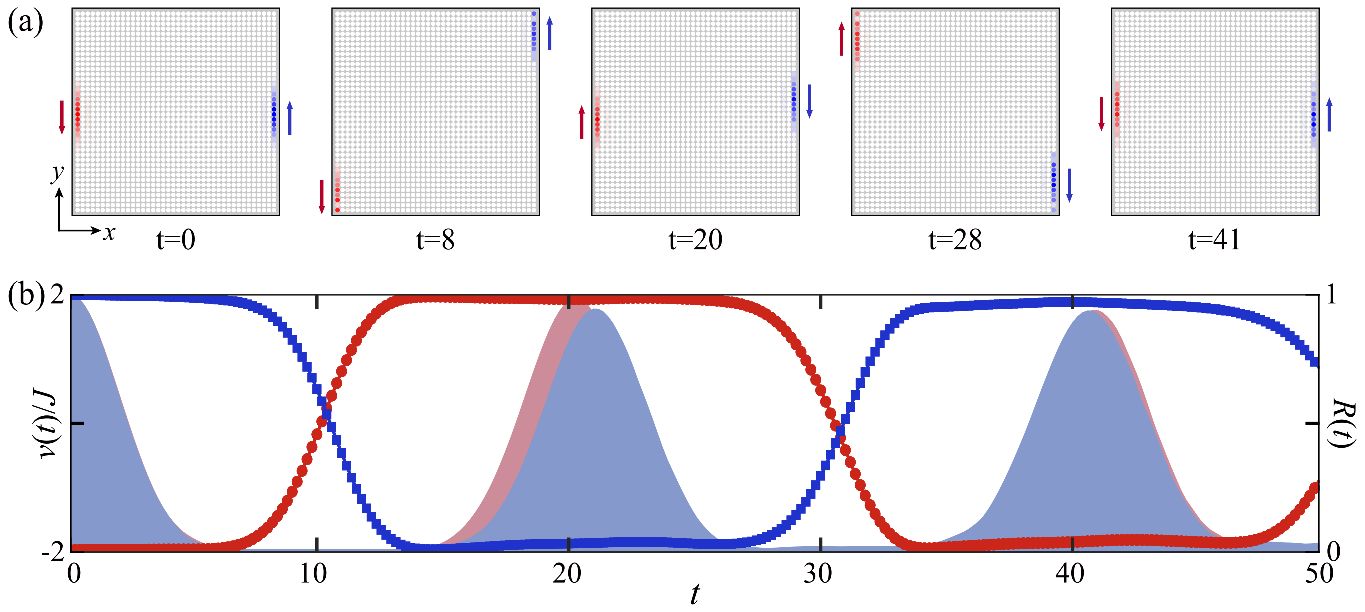

Supplemental Figure 6. (a) Snapshots of counterpropagation along the vertical boundaries and (b) the propagation velocity and return probability for the couplings randomly deviated from the set strengths within the range of $[-5\%, 5\%]$. The Gaussian wave packet of the initial edge state excitation on the left boundary has the momentum $k_y = \pi/2$, and the Gaussian wave packet of the initial edge state excitation on the right boundary has the momentum $k_y = -\pi/2$. Red and blue lines indicate the propagation velocities $\nu(t)$ of the wave packets on the left and right boundaries, respectively. Red and blue areas indicate the return probabilities $R(t)$ of the wave packets on the left and right boundaries, respectively. The lattice size is 40×40, the system parameters are from Fig. 4(a). The unit of time is J^{-1}.

momentum. By contrast, the counterpropagation of in-gap edge states along the parallel boundaries is realized when the excitation of in-gap edge states on the parallel boundaries has the opposite momenta.

Supplemental Figure 6 shows the numerical simulation of the robust counterpropagation of the in-gap edge state excitations. The momentum for the edge state excitation on the left boundary is $k_y = \pi/2$ and the momentum for the edge state excitation on the right boundary is $k_y = -\pi/2$. The parameters of the square lattice are chosen as the topological phase $(P_x, P_y) = (1/2, 0)$ in Fig. 4(a) of the main text. The coupling disorder is set within the range of $[-5\%, 5\%]$. Notably, the counterpropagations along both the left and right boundaries are not obviously affected by the disorder.

* jinliang@nankai.edu.cn

[1] W. A. Benalcazar, B. A. Bernevig, T. L. Hughes, Quantized electric multipole insulators, Science 357 (2017) 61.
[2] F. Liu, H. Y. Deng, K. Wakabayashi, Helical Topological Edge States in a Quadrupole Phase, Phys. Rev. Lett. 122 (2019) 086804.
